# Threat of an influenza pandemic: family physicians in the front line

**DOI:** 10.1186/1471-2296-10-11

**Published:** 2009-02-03

**Authors:** Wim Opstelten, Jim E van Steenbergen, Gerrit A van Essen, Marianne AB van der Sande

**Affiliations:** 1 Dutch College of General Practitioners, Utrecht, The Netherlands, PO Box 3231, 3502 GE Utrecht, The Netherlands; 2 Julius Center for Health Sciences and Primary Care, University Medical Center Utrecht, PO Box 85500, HP. Stratenum 6.109, 3500 AB Utrecht, The Netherlands; 3 Centre for Infectious Disease Control, National Institute for Public Health and the Environment (RIVM), Antonie van Leeuwenhoeklaan 9, 3721 MA Bilthoven, The Netherlands

## Abstract

**Background:**

The chance of an influenza pandemic is real and clinicians should keep themselves informed about the rationale and science behind preventive and therapeutic principles relating to an (impending) influenza pandemic.

**Discussion:**

Vaccination is considered the best prevention in case of a pandemic threat and first choice to contain the impact of a pandemic. Pending the availability of an effective pandemic vaccine, antivirals are likely the only effective agents for prevention and treatment. When an influenza pandemic is impending, all interventions aim to prevent people becoming infected and to suppress replication and transmission of the virus as much as possible. Antivirals will be prescribed to patients with laboratory confirmed pre-pandemic influenza as well as to their contacts (post-exposure prophylaxis) which may delay development of or even prevent a pandemic. During a manifest influenza pandemic, however, there is large-scale spreading of the influenza virus. Therefore, preventive use of antivirals is less efficient to prevent transmission. Delaying the pandemic is then important in order to prevent exhausting public health resources and disruption of society. Thus, during a manifest pandemic everyone with influenza symptoms should receive antivirals as quickly as possible, regardless of virological confirmation. To ensure optimal effectiveness of antivirals and to minimize development of drug resistant viral strains, the use of antivirals for annual influenza should be restrictive. The crucial position of family physicians during an (impending) influenza pandemic necessitates the development of primary health care guidelines on this topic for all countries.

**Summary:**

Family physicians will play a key role in assessing and treating victims of a new influenza virus, and in reassuring the worried well. We outline various possible interventions in the event of an impending and a manifest influenza pandemic, such as non-medial measures, prescription of antivirals, and vaccination, and emphasize the need for pandemic influenza preparedness.

## Background

The chance of an influenza pandemic is real. A pandemic might run a relatively mild course (1956, 1967) or could have disastrous consequences with public health being stretched to its limits and beyond (1918) [1]. Therefore, many countries have drawn up varying strategies to guide policy in the event of an impending or manifest pandemic [2]. Physicians must be informed about preventive and therapeutic strategies in the event of an (impending) influenza pandemic – strategies that are totally different from those for dealing with the annual influenza episodes. This especially applies to family physicians who will be in the front line during a pandemic.

### The emergence of an influenza pandemic

Influenza viruses are continually subject to small mutations. As a result of this so-called *antigenic drift*, annual influenza epidemics emerge, requiring annual vaccination of high risk patients with a yearly adapted vaccine for optimal protection. A totally different situation occurs, when a new influenza virus turns up through a fundamental virus mutation or an exchange of genetic material between a human and an animal influenza virus, against which we have little immunity or cross immunity. This is called an *antigenic shift*. An influenza pandemic can then result through efficient person to person transmission. One cannot predict when and where a pandemic influenza virus will appear and what virulence it will have. However, the risk of human infection with avian influenza is greatest where there is intense contact between people and poultry such as is common in South East Asia. For several years, attention has been focussed on the H5N1 influenza-virus that is circulating, but the emergence of every influenza-A-virus, against which immunity does not, or no longer, exist can lead to a pandemic. Also previous and presently no more on a large-scale circulating viruses, like H2N2, which caused the Asian pandemic in 1957 and against which the majority of the world population lacks protection, could result in pandemic spread.

The World Health Organization (WHO) has defined several phases before and during an influenza pandemic (Table 1) [3]. In the pre-pandemic phases (three to five) there is an increasing degree of pandemic threat. In phase six there is a manifest pandemic. For the H5N1, we are currently in phase three. Every pre-pandemic phase can in principle last for an unlimited period or may be present, locally, for a short period or not at all; phase six ends after the final wave, as soon as the pandemic influenza virus has circulated world-wide and has become a new global epidemic influenza virus.

**Table 1:** WHO pandemic phases

	**Pandemic phases**	**Public health goals**
	**Inter-pandemic period**	
1	No new influenza virus subtypes have been detected in humans. An influenza virus subtype that has caused human infection may be present in animals. If present in animals the risk of human infection or disease is considered to be low.	Strengthen influenza pandemic preparedness at the global, regional, national and sub-national levels.
2	No new influenza virus subtypes have been detected in humans. However, a circulating animal influenza virus subtype poses a substantial risk of human disease.	Minimize the risk of transmission to humans; detect and report such transmission rapidly if it occurs.
	**Pandemic alert period**	
3	Human infection(s) with a new subtype, but no human spread, or at most rare instances of spread to a close contact.	Ensure rapid characterization of the new virus subtype and early detection, notification and response to additional cases.
4	Small cluster(s) with limited human to human transmission but spread is highly localized, suggesting that the virus is not well adapted to humans.	Contain the new virus within limited foci or delay spread to gain time to implement preparedness measures, including vaccine development.
5	Large cluster(s) but human to human spread still localized, suggesting that the new virus is becoming increasingly better adapted to humans, but may not yet be fully transmissible (substantial pandemic risk).	Maximize efforts to contain or delay spread, to possibly avert a pandemic, and to gain time to implement pandemic response measures.
	**Pandemic period**	
6	Pandemic: increased and sustained transmission in general population.	Minimize the impact of the pandemic.
	**Post-pandemic period**	
	Post-pandemic period: return to inter-pandemic period.	Return to inter-pandemic period.

Both non-medical interventions, such as social distancing and public information, as well as medical interventions, such as antivirals and vaccination, are relevant in the prevention and suppression of an influenza pandemic.

### Vaccination

Vaccination is considered the best available prevention in case of a pandemic threat and first choice to contain the impact of a pandemic. A vaccine can be developed once the pandemic virus has been identified, potentially from phase four onwards. From that moment it is expected to take at least four to five months before vaccination can be carried out. As people are lacking any protection against the new influenza virus, repeated vaccination may be necessary for inducing a protective immunological response. It, therefore, may take several weeks from the moment of availability of the vaccine to the stage of clinical protection. Recently, discussions have erupted about how much pre-pandemic vaccination with a related strain could contribute to an effective immunity against a pandemic influenza virus [4]. Pending the availability of an effective pandemic vaccine, antivirals are the only effective agents for the prevention and treatment of infections caused by a pandemic influenza virus.

### Antivirals

Neuraminidase inhibitors are the preferred antivirals to delay or suppress an impending influenza pandemic. Older antivirals such as amantadine are not indicated for use as monotherapy because of an assumed lack of effectiveness. Neuraminidase allows the replicated virus to leave the host cell. By inhibiting this enzyme viral circulation is interrupted, resulting in the infection being contained.

Oseltamivir, administered orally and zanamivir, which is inhaled, are registered both for prevention and treatment. The preventive effectiveness exceeds the therapeutic effectiveness. There are two types of prophylaxis: prophylaxis without previous close contact with an influenza patient (*primary prophylaxis*) and prophylaxis after contact with an influenza patient (*post-exposure prophylaxis*).

Although the preventive and therapeutical effectiveness of neuraminidase inhibitors has been mainly established against the currently circulating human influenza viruses [5,6], their effectiveness regarding a pandemic influenza virus is theoretically plausible [7]. Importantly, neuraminidase inhibitors reduce the concentration of influenza virus in the mucous membrane of the nose and curtail the excretion of the virus without, as far as known, inhibiting the production of virus-specific antibodies [8].

### Policy principles with an (impending) influenza pandemic

The policy in the pre-pandemic phases differs fundamentally from that during a manifest influenza pandemic.

With an impending influenza pandemic, WHO-phases three to five, measures are taken to prevent or delay the development of a pandemic. All interventions are geared towards preventing people becoming infected and to suppress replication and transmission of the virus as much as possible. Interventions can be made in the chain of infection, for example through minimising unprotected contacts with infected poultry, or chance encounters with patients, and through administering antiviral drugs. These drugs will be prescribed to patients with laboratory confirmed pre-pandemic influenza (treatment) as well as to their contacts (post-exposure prophylaxis). This may delay development of or prevent a pandemic [9].

During a manifest influenza pandemic, WHO-phase six, there is large-scale spreading of the influenza virus and, therefore, isolation and preventive use of antivirals are less efficient to prevent transmission. Delaying the pandemic is then important in order to prevent exhausting public health resources and disruption of society. Above all we have to aim at gaining time for developing, testing and producing an effective vaccine. Therefore, the Dutch guideline for family physicians states that during this phase, everyone with influenza symptoms should receive antivirals as quickly as possible, regardless virological confirmation [10]. As a result, patients will be less severely ill, less infectious, ill for a shorter period, and spreading of the virus will thus be delayed. As in this phase the pandemic virus has already rampantly spread, post-exposure prophylaxis is not an efficient intervention anymore.

Primary prophylaxis is not advised for the general public in any of the (pre-)pandemic phases. After stopping the drugs the person remains susceptible to the virus in the absence of immunological protection. Moreover, national stocks of antiviral medicines would likely be inadequate for this policy.

### Implications for today

To ensure optimal effectiveness of antivirals and to minimize the development of oseltamivir-resistant viral strains, the present use of neuraminidase inhibitors should be restrictive. With annual influenza episodes, antivirals have, in practise, only marginal effects on otherwise healthy immunocompetent people, i.e., reducing the average duration of symptoms by roughly one day [6]. Therefore, therapeutic use should be limited to people at high risk of complications. Antivirals as a post-exposure prophylaxis should be recommended only for residents and staff of nursing homes and for severely immunocompromised patients (e.g., those on chemotherapy), who are not, or suboptimally protected by vaccination.

Neuraminidase inhibitors do have a potentially favourable influence on the course of the disease in an individual patient, but are especially valuable, on a population level, in averting or delaying an influenza pandemic, provided antiviral agents are prescribed swiftly and for the correct indication. Not only doctors, but the general public too, must be properly informed about this. Giving concerned citizens antiviral agents in anticipation should be resisted. It risks leading to incorrect use, reduced effectiveness, and/or the emergence of oseltamivir-resistant strains of influenza. Above all, it could give a false sense of security which may delay reporting of symptoms and launching effective interventions [11].

Of particular concern is the emergence of fit and transmissible oseltamivir-resistant influenza A(H1N1) in early 2008, which has by now spread all over the world. The other seasonal influenza strains (A(H3N2 and B) have remained sensitive. This stresses the need to implement real-time monitoring of the effectiveness of antivirals, both during the current phase, as during a potential pandemic.

### Family physicians in the front line

Especially during an (impending) influenza pandemic family physicians will play a key role in signalling pre-pandemic viral infections, assessing and treating victims of the new influenza virus, and reassuring the worried well. This necessitates the development of primary health care guidelines on this topic in all countries [10,12,13]. These should not only comprise medical and non-medical interventions, but also address the logistical problems which will be faced in primary care during an enormously disrupting worldwide influenza outbreak. Obviously, these guidelines will have to harmonise with national and hospital-based policies.

Influenza experts have consistently warned that pandemic influenza is inevitable. Although we do not know when it will strike, we should not linger in urging for pandemic influenza preparedness.

## Competing interests

The authors declare that they have no competing interests.

## Authors' contributions

This debate paper arose from discussions and literature review during the preparation of guidelines for family physicians on the policy during an (impending) influenza pandemic. WO wrote the initial draft. JEvS, GAvE, and MABvdS made substantial contributions and changes.

## Pre-publication history

The pre-publication history for this paper can be accessed here:


